# Microorganisms causing pyogenic spondylitis: Comparison of community and hospital-acquired types

**DOI:** 10.3109/03009734.2012.687406

**Published:** 2012-10-30

**Authors:** Tatsuro Sasaji, Noboru Yamada, Kazuo Iwai

**Affiliations:** Department of Orthopedic Surgery, Fukushima Rosai Hospital, 3-Numajiri, Tsuzura-machi, Uchigo, Iwaki 973-8403, Japan

**Keywords:** Classification, infecting microorganism, pyogenic spondylitis, sepsis

## Abstract

**Staphylococcus aureus:**

Pyogenic spondylitis is a common infectious disease caused by various microorganisms. It is difficult to predict the infecting microorganism at the time of initiation of treatment. Pneumonia is generally clarified into community or hospital-acquired types based on where the infection was acquired, and the infecting microorganisms are different for each type. We retrospectively analyzed 20 cases of pyogenic spondylitis treated in our hospital and categorized the cases into community and hospital-acquired types. We also identified the infecting microorganisms and the rate of sepsis in each type. There were 12 cases of community-acquired and 8 of hospital-acquired infection. The major infecting microorganisms responsible for the community-acquired type were Gram-positive cocci, and those responsible for the hospital-acquired type were methicillin-resistant *Staphylococcus aureus* and Gram-negative bacilli. The rate of sepsis was significantly different for both groups: 16% for the community-acquired type and 75% for the hospital-acquired type. The classification of pyogenic spondylitis based on where the infection was acquired may be useful for predicting which microorganisms are responsible for the disease.

## Introduction

Pyogenic spondylitis is a common orthopedic infectious disease. The incidence of pyogenic spondylitis is increasing in Japan, which is an aging society (1). Treatment of pyogenic spondylitis is generally conservative and comprises of antibiotics, bed rest, and immobilization (2). Antibiotics should be selected according to the sensitivity of the infecting microorganisms. The recommended first-line antibiotics include first and second generation cephalosporins and penicillin, to which Gram-positive cocci are sensitive. However, the infecting microorganisms are sometimes bacteria other than Gram-positive cocci; in such cases, other antibiotics are required. No reports on the classification of pyogenic spondylitis based on the infecting microorganisms are available. For example, pneumonia is generally divided into community and hospital-acquired types based on where the infection was acquired, because the infecting microorganisms are different in each group (3,4). Therefore, we hypothesized that the microorganisms causing pyogenic spondylitis would also differ based on where the infection is acquired, namely, whether the infection is community or hospital-acquired. We retrospectively analyzed the microorganisms causing pyogenic spondylitis among the cases treated in our hospital. The purpose of the present study was to clarify the usefulness of a classification based on where the infection was acquired.

## Patients and methods

All patients were informed that the data from their cases would be used for the study.

### Subjects

The subjects of this study included 20 patients who were treated conservatively for pyogenic spondylitis in our hospital between 2007 and 2011. Eleven patients with pyogenic spondylitis caused by unidentified microorganisms were excluded. We treated no case of pyogenic spondylitis surgically. The diagnosis of pyogenic spondylitis was established by culture of the infected intervertebral disc, laboratory data, including white blood cell count and C-reactive protein level, and magnetic resonance imaging. We analyzed age, sex, the infecting microorganisms, the rate of sepsis, and divided the patients into two types: community-acquired type of pyogenic spondylitis and hospital-acquired type. We analyzed the associated disease in the hospital-acquired type of pyogenic spondylitis. According to the Surviving Sepsis Campaign guideline, the mortality rate of sepsis within the first month of diagnosis was 30% (5). We thought that it was important whether pyogenic spondylitis was accompanied with sepsis or not. We analyzed the infecting microorganisms and merger rate of sepsis in each type. We defined the community-acquired type of pyogenic spondylitis as the disease that occurred in patients at home and the hospital-acquired type as the disease that occurred in patients treated and hospitalized in other departments. Sepsis was defined as a systemic inflammatory response syndrome (SIRS) due to infection. SIRS was diagnosed when two or more of the following criteria were present: high fever or hypothermia, tachycardia, tachypnea, and abnormal white blood cell count (6).

### Statistical analyses

For statistical purposes, the data were analyzed using Fisher's exact probability test to compare the merger rate of sepsis between the types. A probability value of < 0.05 was considered significant. Statistical analyses were performed using GraphPad Prism (GraphPad Software Inc., San Diego, CA, USA).

## Results

A total of 12 male and 8 female patients with a mean age of 70 years (range 16–82 years) were included in the study. The infecting microorganisms were *Staphylococcus aureus* in 5 cases, methicillin-resistant *Staphylococcus aureus* (MRSA) in 3, *Streptococcus agalactiae* in 3, coagulase-negative *Staphylococcus* in 2, *Enterococcus faecalis* in 2, and *Staphylococcus intermedius*, *Pseudomonas aeruginosa*, *Bacillus cereus*, *Campylobacter* species, and *Escherichia coli* in 1 each. Sepsis developed in 8 cases. The community-acquired type of pyogenic spondylitis occurred in 12 cases and the hospital-acquired type in 8. The infecting microorganisms in the community-acquired type were *Staphylococcus aureus* in 5 cases, *Streptococcus agalactiae* in 2, coagulase-negative *Staphylococcus* in 2, and *Staphylococcus intermedius*, *Escherichia coli*, and *Enterococcus faecalis* in 1 each. The infecting microorganisms in the hospital-acquired type were MRSA in 3 cases and *Enterococcus faecalis*, *Pseudomonas aeruginosa*, *Bacillus cereus*, *Campylobacter* species, and *Streptococcus agalactiae* in 1 each (Table I). Gram-positive cocci were found in 10 cases of the community-acquired type. Gram-negative bacilli were found in 4 cases and MRSA in 3 cases of the hospital-acquired type. Sepsis occurred in 2 (16%) cases of the community-acquired type and 6 (75%) cases of the hospital-acquired type (Table II). The rate of sepsis was significantly different between the two types (*P* = 0.019). The associated diseases in the hospital-acquired type of pyogenic spondylitis were alcoholic hepatitis in 3, chronic renal failure treated with hemodialysis in 1, ileus in 1, lung abscess in 1, lumbar injection in another hospital in 1, cardiopulmonary arrest treated in intensive care unit in 1.

**Table I.** Infecting organism in pyogenic spondylitis.

	Infecting microorganism
Community-acquired type (12 cases)	*Staphylococcus aureus* *Streptococcus agalactiae* Coagulase-negative *Staphylococcus* *Staphylococcus intermedius* *Escherichia coli* *Enterococcus faecalis* (Gram-positive coccus: 10 cases)
Hospital-acquired type (8 cases)	Methicillin-resistant *Staphylococcus aureus* *Enterococcus faecalis* *Pseudomonas aeruginosa* *Bacillus cereus* *Campylobacter* species *Streptococcus agalactiae* (MRSA: 3 cases; Gram-negative bacilli: 4 cases)

**Table II.** Comparison of the rate of sepsis.

	Community-acquired type (12 cases)	Hospital-acquired type (8 cases)
The merger rate of sepsis (sepsis cases)	16% (2 cases)	75% (6 cases)

*P* = 0.019 compared with each type (Fisher's exact probability test).

## Discussion

The treatment of pyogenic spondylitis is generally conservative and comprises of antibiotics, bed rest, and immobilization (2). Appropriate antibiotic selection is important until the sensitivity of the infecting microorganism can be established by culture sensitivity testing. *Staphylococcus aureus* is reportedly the most common microorganism causing pyogenic spondylitis (2). On the other hand, microorganisms other than *Staphylococcus aureus* have also been reported to cause this infection (2). It was unclear what factor was responsible for the difference in infecting microorganisms. We classified pyogenic spondylitis based on where the infection was acquired in the present study. The major infecting microorganisms were Gram-positive cocci in the community-acquired type and Gram-negative bacilli and MRSA in the hospital-acquired type. Thus, classification based on where the infection was acquired may be useful for predicting the identity of the infecting microorganisms.

Since the development of chemotherapy, the prognosis of pyogenic spondylitis is generally good. Generalized sepsis indicates a primary source of infection other than the spine (2). However, sepsis occurred in some cases in the present study. Mortality associated with sepsis is unacceptably high, and rapid management is critical (5). In the present study, the rate of sepsis was higher in the group with hospital-acquired type of pyogenic spondylitis. Immediate prescription of wide-spectrum antibiotics to which Gram-negative bacilli and MRSA are sensitive is essential for the hospital-acquired type of pyogenic spondylitis.

No reports on the classification of pyogenic spondylitis based on the infecting microorganisms are available. On the basis of present study, we assumed that classification based on where the infection was acquired was useful for predicting the identity of the infecting microorganism and for selecting the appropriate first-line antibiotics therapy. However, the number of cases was small in the present study. Further studies with a large number of pyogenic spondylitis patients would be necessary to address this question.
